# Validation of self-reported family history of myocardial infarction using nationwide health care data

**DOI:** 10.1007/s10654-026-01399-x

**Published:** 2026-05-16

**Authors:** Agnes Wahrenberg, Karin Leander, Henrike Häbel, Patrik K. E. Magnusson, Ralf Kuja-Halkola, Göran Bergström, Lars Lind, Emil Hagström, Gunnar Engström, Tomas Jernberg, Stefan Söderberg, Carl Johan Östgren, Per Svensson

**Affiliations:** 1 Department of Clinical Science and Education, Södersjukhuset, Karolinska Institutet, Sjukhusbacken 10, 11883 Stockholm, Sweden; 2 Institute of Environmental Medicine, Karolinska Institutet, Stockholm, Sweden; 3 Department of Learning, Informatics, Management and Ethics, Karolinska Institutet, Stockholm, Sweden; 4 Department of Medical Epidemiology and Biostatistics, Karolinska Institutet, Stockholm, Sweden; 5 Institute of Medicine, Sahlgrenska Academy, University of Gothenburg, Gothenburg, Sweden; 6 Department of Medical Sciences, Clinical Epidemiology, Uppsala University, Uppsala, Sweden; 7 Uppsala Clinical Research Center, Uppsala University, Uppsala, Sweden; 8 Department of Clinical Sciences Malmö, Lund University, Malmö, Sweden; 9 Department of Clinical Sciences, Danderyd University Hospital, Karolinska Institutet, Stockholm, Sweden; 10 Department of Public Health and Clinical Medicine, Umeå University, Umeå, Sweden; 11 Department of Health, Medicine and Caring Sciences, Linköping University, Linköping, Sweden; 12 Centre for Medical Image Science and Visualization (CMIV), Linköping University, Linköping, Sweden

**Keywords:** Family history, Medical history taking, Myocardial infarction, Cardiovascular risk

## Abstract

Self-reported family history of myocardial infarction (MI) is frequently used to assess cardiovascular risk. However, validation of self-reported family history has often been limited to small samples, and to death-certificates or self-reported medical history as reference. This study investigated the accuracy of self-reported family history of MI in a population-based cohort, using register data as reference. We included 25,302 participants from the Swedish CArdioPulmonary bioImage Study (SCAPIS) with sufficient register coverage. Self-reported family history of MI in parents and siblings was obtained from the SCAPIS questionnaire. Relatives were identified in the Swedish Multi-Generation Register and occurrences of MI were retrieved from the Swedish National Patient Register and Cause of Death Register. Cohen’s κ, sensitivity, specificity, and predictive values for self-reports were calculated using register-derived diagnoses as reference. The mean age of participants was 57.5 years. The overall agreement of self-reported and register-verified family history was moderate. Reporting family history of MI in any parent or sibling yielded a κ of 0.491, a sensitivity of 57.6% and a specificity of 89.0%. The positive and negative predictive values were 73.9 and 79.6%, respectively. The accuracy of reported maternal and paternal MI were similar, however, reports of early-onset disease showed lower overall κ. Male participants consistently reported family history less accurately than females, and participants with a university degree reported family history more accurately than those without a university education. Compared with register data, self-reported family history of MI underestimates the prevalence of, especially early-onset, MI in close relatives.

**Supplementary Information:**

The online version contains supplementary material available at 10.1007/s10654-026-01399-x.

## Introduction

Having a family history of myocardial infarction (MI) is an established risk factor for incident cardiovascular disease [1–4]. Self-reported family history of MI from interviews or questionnaires is commonly used in clinical routine and in research settings [4, 5], however, self-reports of medical conditions are not always consistent with medical records [5–7]. Previous validations of self-reported family history of MI and cardiovascular diseases have been limited to smaller samples, and have used relatives’ own health-reports, local medical records or death certificates as reference, with reported moderate sensitivity [5, 8–12]. The accuracy of self-reported family history also varies across diagnoses, as reports of familial cardiovascular risk factors have shown to have lower sensitivity than for familial MI [9, 11].

Misclassification of family history can lead to bias in research about its clinical significance. The increase in cardiovascular risk associated with a family history of MI cannot fully be explained by a genetic risk score or traditional cardiovascular risk factors and thus deems further investigation [13–15]. National quality registers and governmental registers are now increasingly used for research, and a detailed family history of a variety of diseases can be retrieved from centralized registers [16–18]. To our knowledge, no previous study has determined the accuracy of a self-reported family history of MI compared with register-based health care data. Furthermore, the reliability of national patient registers in the Nordic countries has been deemed high for diagnoses such as MI [19, 20]. We therefore assessed the agreement of a self-reported family history of MI from a Swedish population cohort using national register data as reference.

## Methods

### Study participants and setting

The Swedish CArdioPulmonary bioImage Study (SCAPIS) is a population-based cohort recruited randomly from the Swedish census register [21]. Individuals aged 50–64 years were invited to participate at six Swedish university hospitals between 2013 and 2018. Only those with sufficient Swedish language proficiency were included, and all provided written informed consent. Ultimately, 30,154 participants (50.3% of the invitees) were included in the SCAPIS cohort and underwent examinations comprising a detailed questionnaire, physical examination, laboratory tests and cardiac imaging. The full examination protocol is provided elsewhere[21]. Study participants were asked to report on their health, medications, lifestyle habits and social determinants, as well as on family history of several diseases in the questionnaire. Family history of MI was assessed with a main question; *“Has your father, mother or sibling ever suffered from or died from a heart attack?”*, and two follow up questions*; “If yes, please indicate all that apply: My mother, my father, one or more siblings, Don’t know/Prefer not to answer”* and *“If yes, how old were they at the time (youngest age)?”*. Missing answers and the answer “*Don’t know/Prefer not to answer”* on the main question were, for the purpose of validating reported family history in this study, treated as no reported family history. We included participants from SCAPIS consenting further register linkage, with no re-used personal identification numbers (PIN) and both parents identifiable in the Swedish Multi-Generation Register (MGR).

### Register linkage

Data on SCAPIS participants were linked to Swedish national registers by means of the PIN, issued to all residents by the Swedish Tax Agency [22]. Although rare, some PINs are reused, such as when an immigrated resident receives the PIN of a deceased individual [22]. The PINs that were marked as reused were excluded from further analyses. Parents, full siblings and half-siblings were identified in the MGR. This register is provided by Statistics Sweden and contains information on family relationships for the majority of individuals born in Sweden from 1932, residing in Sweden from 1962 onward [23]. The information is mainly based on census data, and, in some cases, court acknowledgements of parentage. Data on relatives were subsequently linked to the National Patient Register (NPR) and the Cause of Death register, to retrieve registered diagnoses of MI. Age of disease onset in relatives was used to construct age-dependent definitions of early-onset disease. The NPR contains information on diagnoses and procedures registered in inpatient care as well as from hospital-based outpatient care. Coverage of the Swedish population’s diagnoses from inpatient care has increased from the implementation in 1964, reaching nearly complete coverage of public caregivers from the late 1980s. The sensitivity for a diagnosis of MI in NPR has been reported above 90% [20]. Income data for the SCAPIS recruitment year were retrieved from Statistics Sweden and were divided into income-year and gender adjusted quintiles. A more detailed description of the registers used in this study is presented in the Supplementary material.

### Definitions of family history

Registered diagnoses of fatal or non-fatal MI in the NPR or Cause of Death register of relatives were used to construct register-based definitions for family history, corresponding to the self-reports in the SCAPIS-questionnaire. As the questionnaire implicitly asked for cases of “heart attack”, we did not include other cardiovascular outcomes, such as stable angina or elective revascularization procedures. Only the primary diagnosis of registered hospital admissions and the underlying and contributory causes of death were used for validation. The diagnoses considered according to International Classification of Diseases (ICD) systems are provided in Supplementary Table S1. A first registered MI occurring before 55 years in male relatives and before 65 years in female relatives was considered early-onset, according to current European Society of Cardiology guidelines [24, 25]. Age at the first MI was also used to validate a post-hoc constructed SCAPIS variable considering MI before the age of 60 in any parent.

### Statistical methods

All data management and statistical analyses were conducted using STATA, version 16.1 (StataCorp., College Station, TX, USA). Characteristics of the SCAPIS individuals were summarized as frequencies and proportions for categorical variables, and as the mean and standard deviation (SD) for numerical variables. The standardized difference was calculated for each characteristic according to family history status as the difference between group means divided by the pooled standard deviation for continuous variables and for categorical variables, by assessing the multivariate Mahalanobis distance between group-specific proportion vectors [26]. Cohen’s κ was used to estimate the agreement of self-reported and register-based family history, in which the proportion of observed agreement is related to the expected agreement by chance, using an analytical method to estimate 95% confidence intervals (CI) [27]. The sensitivity and specificity were calculated as the rates of true positives and true negatives of cases identified in the questionnaire, respectively. The positive predictive value (PPV) was calculated as the probability of register-verified family history given a reported history, and conversely, the negative predictive value (NPV) was calculated as the probability of no registered family history given no reported history. Due to the nature of the follow-up questions in the SCAPIS questionnaire, participants reporting more than one afflicted relative were only recorded with one age-at-onset variable, regardless of the number of relatives reported, which then could not reliably be assigned to a specific relative. As early-onset disease has been deemed relevant at different ages in men and women, analyses of sex- and age-restricted family history were only assessed in a subset of participants only reporting one afflicted relative. The post-hoc constructed SCAPIS variable of parental MI before the age of 60 years was, however, validated in the whole study sample, as the same age cut-off was used for both sexes. Subgroup analyses were performed for sex, self-reported educational attainment and year- and gender-adjusted quintiles of income. A restricted analysis of participants with parents born in 1930 or later was also conducted as a sensitivity analysis to explore temporal differences in reporting accuracy.

### Ethical considerations

The implementation of SCAPIS was approved by the Regional Ethical Board at Umeå University (2010-228-21M) and this validation was approved by the Swedish Ethical Review Authority (2021-02951, 2022-04143-02). Due to the sensitive nature of data, it will not be made available to other researchers, in concordance with prevailing ethical permits.

## Results

**Fig. 1:**
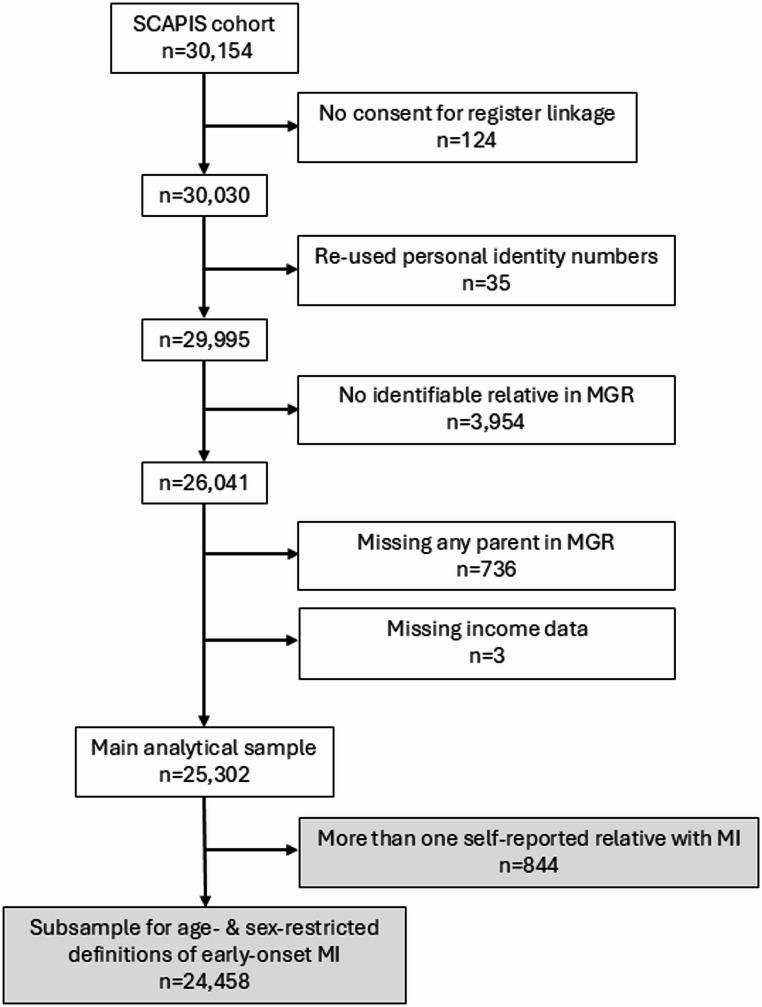
Inclusion process of SCAPIS participants in the main sample and a subsample for which only one relative afflicted by myocardial infarction was reported. MGR the Swedish multi-generation register, MI myocardial infarction, SCAPIS the Swedish CArdioPulmonary bioImage Study

In total, 25,302 individuals were ultimately included from SCAPIS. The inclusion process is shown in Fig. 1. The median age was 57.4 years and 51% of participants were female. From the questionnaire, 6905 participants (27.3%) reported a family history of MI in any parent or sibling, whereas an MI-diagnosis in relatives from registers was retrieved for 8855 participants (35.0%). Characteristics according to self-reported and register-verified family history of MI are presented in Table 1 and according to sex in Supplementary Table S2. Characteristics of individuals excluded due to missing information in MGR are described in Supplementary Table S3. Participants reporting a family history of MI were more often female and slightly older, and they reported more commonly a previous history of MI, coronary revascularization and diabetes, as well as higher rates of antihypertensive and lipid-lowering therapy use. Similar differences in the distribution of cardiovascular risk factors, diseases and treatments were observed between those with and without a register-verified MI in any relative.

**Table 1:** Characteristics of SCAPIS participants with both parents identifiable in the Swedish multi-generation register

	Total sample	Self-reported MI in parent or sibling		Standardized difference	Register-verified MI in parent or sibling		Standardized difference
		No	Yes		No	Yes	
	n = 25,302	n = 18,397	n = 6905		n = 16,447	n = 8855	
Female sex	12,953 (51.2%)	9088 (49.4%)	3865 (56.0%)	0.132	8414 (51.2%)	4539 (51.3%)	0.002
Age	57.5 (4.4)	57.3 (4.4)	58.0 (4.3)	− 0.157	57.1 (4.4)	58.2 (4.3)	− 0.258
Smoking				0.062			0.060
Former smoker	8941 (35.3%)	6302 (34.3%)	2639 (38.2%)		5671 (34.5%)	3270 (36.9%)	
Never smoked	12,717 (50.3%)	9285 (50.5%)	3432 (49.7%)		8436 (51.3%)	4281 (48.3%)	
Current smoker	2813 (11.1%)	2061 (11.2%)	752 (10.9%)		1818 (11.1%)	995 (11.2%)	
Missing	831 (3.3%)	749 (4.1%)	82 (1.2%)		522 (3.2%)	309 (3.5%)	
Education, highest completed level				0.017			0.073
Degree from university	11,215 (44.3%)	8063 (43.8%)	3152 (45.6%)		7490 (45.5%)	3725 (42.1%)	
History of myocardial infarction	376 (1.5%)	207 (1.1%)	169 (2.4%)	0.097	170 (1.0%)	206 (2.3%)	0.103
History of PCI or CABG	257 (1.0%)	125 (0.7%)	132 (1.9%)	0.107	110 (0.7%)	147 (1.7%)	0.094
Antihypertensive medication	4939 (19.5%)	3290 (17.9%)	1649 (23.9%)	0.134	2906 (17.7%)	2033 (23.0%)	0.136
Lipid lowering medication	1926 (7.6%)	1172 (6.4%)	754 (10.9%)	0.155	1012 (6.2%)	914 (10.3%)	0.155
History of diabetes	1002 (4.0%)	660 (3.6%)	342 (5.0%)	0.062	566 (3.4%)	436 (4.9%)	0.076

Categorical variables reported as n (%), continuous variables reported as mean (SD)

*CABG* coronary artery bypass grafting, *IQR* interquartile range, *MI* myocardial infarction, *PCI* percutaneous coronary intervention

**Fig. 2:**
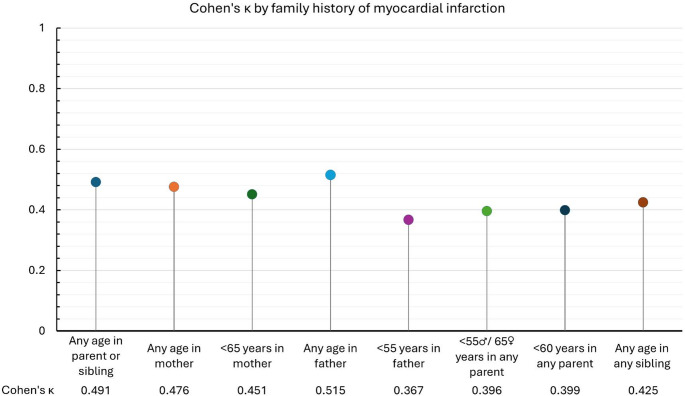
Cohen’s κ according to each definition of family history of myocardial infarction

Cohen’s κ is visualised in Fig. 2, and reported along with sensitivity, specificity, PPV and NPV for each definition of family history in Table 2. For a self-reported family history MI in any parent or sibling, general agreement measured by κ was moderate (κ = 0.491, 95% CI 0.480–0.503) along with a sensitivity of 57.6% and specificity of 89.0%. The PPV and NPV for MI in any relative were 73.9 and 79.6%, respectively. Generally, the specificity of self-reported family history was high, ranging from 89.0% at lowest (MI at any age in any parent or sibling) to 99.1% at highest (MI at any age in any sibling and MI < 65 years in mother). The κ for maternal history of any age and early-onset MI were largely similar, whereas the κ for paternal history was substantially lower for early-onset disease than for disease at any age of onset. The sensitivity for a reported history of MI in any sibling was the lowest observed, at 33.0% (95% CI 30.4–35.7%).

**Table 2:** Measures of agreement of self-reported family history of myocardial infarction in parents and siblings compared to register data

	Self-reported/register confirmed				Cohen’s κ (95% CI)	Sensitivity (95% CI)	Specificity (95% CI)	Positive predictive value	Negative predictive value
	− / −	+ / −	− / +	+ / +					
MI at any age in parent or sibling	14,643	1804	3754	5101	0.491 (0.480–0.503)	57.6% (56.6–58.6%)	89.0% (88.5–89.5%)	73.9% (72.8–74.9%)	79.6% (79.0–80.2%)
MI at any age in mother	21,637	609	1751	1305	0.476 (0.459–0.494)	42.7% (40.9–44.5%)	97.3% (97.0–97.5%)	68.2% (66.0–70.3%)	92.5% (92.2–92.8%)
MI < 65 years in mother*	23,944	215	146	153	0.451 (0.404–0.499)	51.2% (45.4–57.0%)	99.1% (99.0–99.2%)	41.6% (36.5–46.8%)	99.4% (99.3–99.5%)
MI at any age in father	17,575	1777	2461	3489	0.515 (0.502–0.528)	58.6% (57.4–59.9%)	90.8% (90.4–91.2%)	66.3% (65.0–67.5%)	87.7% (87.3–88.2%)
MI < 55 years in father*	23,580	547	126	205	0.367 (0.330–0.404)	61.9% (56.5–67.2%)	97.7% (97.5–97.9%)	27.3% (24.1–30.6%)	99.5% (99.4–99.6%)
MI before age 55♂/65♀ in any parent*	23,076	757	262	363	0.396 (0.367–0.426)	58.1% (54.1–62.0%)	96.8% (96.6–97.0%)	32.4% (29.7–35.2%)	98.9% (98.7–99.0%)
MI < 60 years in any parent	23,294	1103	361	544	0.399 (0.374–0.423)	60.1% (56.8–63.3%)	95.5% (95.2–95.7%)	33.0% (30.8–35.4%)	98.5% (98.3–98.6%)
MI at any age in any sibling	22,052	192	835	412	0.425 (0.397–0.454)	33.0% (30.4–35.7%)	99.1% (99.0–99.3%)	68.2% (64.3–71.9%)	96.4% (96.1–96.6%)

*Minus (−)* absence of MI, *plus (+)* presence of MI, *CI* confidence interval, *MI* myocardial infarction

*Analysis performed in subset of participants with no more than one self-reported family member afflicted by myocardial infarction

Female participants reported a family history of MI significantly more often than male participants. Generally, any family history of MI reported by a female respondent demonstrated higher measures of κ and sensitivity compared to the same history reported by a male respondent, although the differences were smaller for age-restricted disease. Measures of agreement of reported history according to sex are presented in Supplementary Table S4. In participants with a university degree, measures of agreement were generally higher, although the differences were small for age-restricted disease as seen in Supplementary Table S5. There was a trend of higher agreement across year- and gender-adjusted income quintiles for family history definitions without age restrictions, presented in Supplementary Table S6.

## Discussion

We have measured the accuracy of self-reported family history of MI in a contemporary, population-based cohort of middle-aged individuals. Using Swedish national registers of kinship and records from hospital discharges and causes of death, we provide a reliable and comprehensive basis for confirmation of disease in relatives. Generally, all definitions of self-reported family history of MI demonstrated moderate agreement with register-verified data, with κ’s from 0.367 to 0.515, and sensitivities ranging from 33.0% at lowest to 61.9% at best. The highest sensitivity and PPV was observed for the simplest questionnaire item of MI without age-restriction in any parent or sibling, as well as for paternal and parental history of MI.

In previous validation studies, the sensitivity of self-reported family history of MI has ranged between 67 and 87% [5, 8–12]. This study is, to our knowledge, the first to report on the accuracy of self-reported family history of MI using national registers as reference. While specificity remained high, the sensitivity and PPV deteriorated when assessing age-specific family history. Similarly, in data from the Framingham Heart Study the PPV for a self-reported history of early-onset MI before the age of 55 in fathers was notably low at 28%, however, the prevalence of such disease was low in that sample [9]. When the validation definition was widened to paternal MI at any age the PPV rose to 54%, indicating recall difficulties of the age of disease-onset in relatives in that cohort. Considering early-onset MI in fathers in this study, the agreement between self-reported and register-based history as measured with κ was lower than for paternal MI without age-restrictions, however, the sensitivity was higher and the PPV was lower. This suggests that there was more inaccurate over-reporting of early-onset paternal MI. The same trend was observed also for early-onset and ever-occurring maternal MI, albeit with a higher PPV. This is of special importance, as a family history of early-onset MI has been shown to infer higher cardiovascular risk than a history of MI in any age [16].

Across most definitions of family history, female respondents reported more accurately according to registers than male counterparts. In a survey on awareness of family history of diseases in general from the US Centers for Disease Control and Prevention, males were significantly less likely to collect family history information in general and for type 2 diabetes, although knowledge of family history was deemed important for most respondents [28]. Sex differences in reporting family history, specifically the lower likelihood of men to report such information, have been observed for a family history of cancer [29]. To our knowledge, however, this has not been demonstrated for a family history of MI. Across year- and gender adjusted income quintiles and educational attainment, there were trends for higher agreement and sensitivity for several definitions of family history. This has previously been shown for self-reported personal history of cardiovascular diseases [30]. Individuals with a personal history of MI were more likely to report a family history, and to do so correctly (data not shown). While this may reflect familial clustering of cardiovascular disease, it may also be due to increased awareness of familial disease.The sensitivity of a self-reported history of MI in any sibling was distinctly low, similarly to a previous assessment of reported family history of coronary heart disease including MI and revascularization therapies, where the accuracy of reported history was the lowest for siblings [11]. This is especially noteworthy as a history of MI in siblings has shown to be a stronger risk factor for incident MI than parental history of the same [3, 8]. The sensitivity of reported MI in spouses showed the highest accuracy in a study where the relatives’ own health information were the basis for validation, indicating that information on the next of kin is the easiest recollected, also reflected by how the accuracy of family history information on second-degree relatives are even lower [10, 11].

Possibly, some of the misclassifications of family history may arise from uncertainty of medical language and diagnostic criteria. Again, in the Framingham Heart Study, the PPV of reported paternal history increased to 86% when both MI and coronary heart disease at any age of onset were included as correct diagnoses in the validation. Possibly, as coronary diseases other than a definite MI, such as angina pectoris and sudden cardiac death, may be considered as different expressions of the same atherosclerotic disease, these diagnoses may be incorrectly reported in family history questionnaires. However, when unstable angina with any revascularization intervention during the hospital stay, as well as cardiovascular death were included in our validation analyses, sensitivity of self-reported disease in any relative was not improved (data not shown). Because our analysis focused solely on family history of MI, it remains uncertain whether misreporting patterns differ for other cardiovascular outcomes. Furthermore, awareness and perception of disease in general and MI in particular among relatives may vary over time, i.e. due to improvement in diagnostics and increased public awareness. Whereas such differences in reporting accuracy were not assessed in this study, this could contribute to some degree of misclassification.

There are many cardiovascular risk assessment tools built on scores aggregating cardiovascular risk factors. While some of them include family history of cardiovascular disease as a risk factor, the additional value of family history alongside conventional risk factors has been questioned [25, 31]. Misreporting on family history could potentially lead to the underestimation of the relative importance of family history in assessing CVD risk and subsequently lead to failure to initiate preventive treatment measures [25]. While our study shows only moderate accuracy of self-reported family history of MI when compared with register data, it should be emphasized that self-reported family history remains an important tool in clinical practice. It is readily available at the time of patient encounter at low cost, and even with modest misclassification it provides valuable information for risk stratification that is also additive to other sources of inherited risk, such as polygenic risk scores [32, 33].

### Limitations

While the findings of this study provide valuable insights into the validity of self-reported family history of MI, there are also important limitations to be addressed. Firstly, national registers were used as the gold standard for identifying occurrences of MI in relatives. Whereas the Swedish NPR has shown excellent validity of MI diagnoses, coverage is not complete. The sensitivity for a diagnosis in the Cause of Death register is reportedly slightly lower than for hospital discharge data in NPR, despite not taking into account the declining rates of autopsies in Sweden [34]. Reviewing medical records could have provided even greater accuracy of the reference material, however, this was neither practical nor possible for this large-sample validation effort. Secondly, as the coverage of registered diagnoses in the NPR is lower for hospitalizations before the 1980s, it cannot be ruled out that our reference material underestimates the actual prevalence of MI in some of the older relatives. While the SCAPIS index cohort and their siblings would have sufficient coverage in the NPR, the median birth year of the parents of included SCAPIS participants was around the 1930s. Considering very early-onset occurrences of MI in the ages 40–55 years, the corresponding coverage of the NPR is not complete. Whereas the metropolitan regions of central and southern Sweden had adequate coverage in the early 1970s, smaller counties in the western and northern Sweden had sufficient coverage about 10–15 years later [35]. Although less inhabited, the incidence and mortality of MI is higher in these regions [36] and incomplete coverage could affect the measured sensitivity and specificity for early-onset and non-fatal disease. However, as the coverage of fatal events has been comprehensive nationally since the 1950s, and the coverage of events recurring later in time and therefore later in life has increased gradually, the effect on sensitivity and specificity for life-time disease in parents is expected to be small. In a sensitivity analysis we excluded participants with a parent born before 1930, in which the agreement and sensitivity for MI at any age of onset in a relative were modestly higher, however, estimates for early-onset disease were largely unchanged or only slightly lower. The lack of improved agreement for early-onset disease after excluding the oldest individuals suggests that insufficient register coverage is unlikely to be a major contributing factor. The distribution of age and birth year of relatives is described in Supplementary Table S7, and agreement measures of the restricted post-hoc cohort are shown in Supplementary Table S8. Thirdly, a subset of SCAPIS participants had either no relative or only one parent retraceable in the MGR. This is usually due to immigration; however, we cannot exclude that the reporting pattern of family history differs in this subgroup. Fourthly, for the purpose of validation, missing questionnaire data on family history was replaced with no history. However, in a review of possible biases of self-reported family history, Silberberg et al. observed that the conversion of “Don’t know”-answers to “No family history” did not alter measures of agreement [37].

### Conclusions

We conclude that the accuracy of a self-reported family history of MI in this sample is only moderate. Furthermore, we determine that self-reported family history underestimates the prevalence of familial MI according to registers. This indicates that clinical inquiries, especially for an early-onset family history of MI, may also be underestimated. Additionally, the effects of family history on general cardiovascular risk previously derived from studies on self-reported family history may also be biased. It is still unknown whether an improvement in reporting of family history would alter risk assessments or clinical outcomes significantly. While a reported family history of MI remains a clinically meaningful and well-established cardiovascular risk factor, its absence does not rule out true familial risk. National register data may serve as a useful complementary source of family history information, particularly in epidemiological studies where a more accurate assessment of familial risk is needed.

## Supplementary Information

Below is the link to the electronic supplementary material.

Supplementary Material 1

## Data Availability

Due to the sensitive nature of processed data, it cannot be made publicly available. The SCAPIS organization however have application procedures for sharing of data from the original SCAPIS cohort.
