# Supplementary material for: Validation of self-reported family history of myocardial infarction using nationwide health care data
Source: Eur J Epidemiol. 2026 May 16;41(6):765–73. doi: 10.1007/s10654-026-01399-x (PMC13424489; doi:10.1007/s10654-026-01399-x)
Supplement: Supplementary file 1 — Supplementary Material 1 [file 10654_2026_1399_MOESM1_ESM.docx]

Supplementary material

Validation of self-reported family history of myocardial infarction using nationwide health care data

Agnes Wahrenberg^[[1]](#footnote-1)^, Karin Leander^[[2]](#footnote-2)^, Henrike Häbel^[[3]](#footnote-3)^, Patrik K.E. Magnusson^[[4]](#footnote-4)^, Ralf Kuja-Halkola^4^, Göran Bergström^[[5]](#footnote-5)^, Lars Lind^[[6]](#footnote-6)^, Emil Hagström^[[7]](#footnote-7)^ Gunnar Engström^[[8]](#footnote-8)^, Tomas Jernberg^[[9]](#footnote-9)^, Stefan Söderberg^[[10]](#footnote-10)^, Carl Johan Östgren^[[11]](#footnote-11),^^[[12]](#footnote-12)^, Per Svensson^1^

Supplementary information on registers

**The Swedish Multi-Generation Register**

Statistics Sweden organizes the Multi-Generation Register (MGR) of biological and adoptive relatives of Swedish residents[1]. It covers a majority of individuals born on January 1^st^, 1932, or later that have resided in Sweden at least once since January 1^st^, 1961. The introduction of the Swedish personal identification number (PIN) in 1947 enabled the establishment of MGR, when personal identity records including the PIN of relatives was established for children aged 15 or younger at this time. Since the population census management was automatized in 1961, the MGR is based on the Swedish census instead of personal acts. As the census was computerized in 1991, personal records of individuals deceased before 1991 had to be manually reconstituted. Currently, the MGR is constructed hierarchically from the Swedish census, personal identity records and a national register of births. Paternity is established automatically if the child’s mother is married or recently widowed at the time of birth. In other cases, paternity has to be personally reported to the National Tax Agency perinatally, or later through court order. Coverage of information on mothers is nearly complete for individuals born in 1947 and onwards, whereas information on fathers is estimated to be 95% complete for individuals born after 1947 and 98% for those born after 1961. Missing information on mothers may be due to incorrectly registered PINs of the mother, or very low as well as high age of the mother at the birth of the index person. Overall, the register is considered of high quality, however, with limitations above that need to be considered when interpreting register-based family studies.

**The Swedish National Patient Register**

The Swedish National Board of Health of Welfare manages the National Patient Register (NPR). The register contains information on diagnoses from inpatient care for national statistics and health monitoring as well as for quality assurance and research. At the start in 1964, the register collected inpatient information from six of Sweden’s 21 county councils. Coverage increased gradually until complete national coverage in 1987[2]. Since 2001, specialized and hospital-based outpatient care was added, however, primary and municipal care is not reported in NPR. The temporal coverage of NPR before 1987 varies across counties. Whereas Uppsala is covered since 1964, full regional coverage was attained in 1970-1972 for the metropolitan regions of Stockholm, Malmö/Lund and Gothenburg. These comprise four of the six recruitment centres for SCAPIS. For Umeå and Linköping sites, full coverage was achieved in 1984 and 1987, respectively.

**The Swedish Cause of Death Register**

Also managed by the National Board of Health and Welfare, the Cause of Death Register collects diagnoses from death certificates and cause-of-death reports issued by a physician or coroner since 1952[3]. The underlying cause of death, defined as the disease or injury which initiated the chain of events directly leading to death, as well as multiple, contributing causes are reported and classified according to reporting standards of the World Health Organization. In less than 1% of deaths, no cause-of-death certificate is provided, and the corresponding cause is then coded as non-determinable in the register. Of all deaths in the register, 96% have a recorded cause of death. Generally, the quality of reported causes of death varies across diagnoses and diagnostic groups, however, for ischemic heart disease the accuracy of the reported cause of death in the register as compared to case summaries from the corresponding hospitalization has been reported to be very high. While the autopsy rates have diminished during the last decades in Sweden, the improvement in diagnostics during the same time is estimated to account for this decline[4, 5]

Supplementary Table S1. *ICD-codes considered in national registers according to current and historical ICD-systems.*

| *Familial disease of interest* | *ICD-10* | *ICD-9* | *ICD-8* | *ICD-7* | *ICD-6* | *Register* |
| --- | --- | --- | --- | --- | --- | --- |
| Fatal or nonfatal myocardial infarction | I21 – I22 | 410 | 410.00 – 410.99 | 4201 | 4201 | Inpatient, Cause of death |

********Abbreviations: ICD – International Classification of Diseases*

Supplementary Table S2. *Characteristics of included SCAPIS subjects according to sex.*

|  | Females | Males | Total | Standardized difference |
| --- | --- | --- | --- | --- |
|  | **N=12,953** | **N=12,349** | **N=25,302** |  |
| Age | 57.4 (53.7 – 61.2) | 57.5 (53.6 – 61.3) | 57.4 (53.7 – 61.2) | -0.003 |
| Smoking |  |  |  | 0.169 |
| Former smoker | 5,049 (39.0%) | 3,892 (31.5%) | 8,941 (35.3%) |  |
| Never smoked | 6,040 (46.6%) | 6,677 (54.1%) | 12,717 (50.3%) |  |
| Current smoker | 1,498 (11.6%) | 1,315 (10.6%) | 2,813 (11.1%) |  |
| Missing | 366 (2.8%) | 465 (3.8%) | 831 (3.3%) |  |
| Education, highest completed level |  |  |  | 0.198 |
| Degree from university | 6,351 (49.0%) | 4,864 (39.4%) | 11,215 (44.3%) |  |
| History of myocardial infarction | 91 (0.7%) | 285 (2.3%) | 376 (1.5%) | 0.136 |
| History of PCI or CABG | 43 (0.3%) | 214 (1.7%) | 257 (1.0%) | 0.142 |
| Antihypertensive medication | 2,319 (17.9%) | 2,620 (21.2%) | 4,939 (19.5%) | 0.092 |
| Lipid lowering medication | 748 (5.8%) | 1,178 (9.5%) | 1,926 (7.6%) | 0.148 |
| History of diabetes | 371 (2.9%) | 631 (5.1%) | 1,002 (4.0%) | 0.119 |
| Self-reported family history of myocardial infarction, parent or sibling | 3,865 (29.8%) | 3,040 (24.6%) | 6,905 (27.3%) | 0.118 |
| Register-based family history of myocardial infarction, parent or sibling | 4,539 (35.0%) | 4,316 (35.0%) | 8,855 (35.0%) | 0.002 |
| Self-reported family history of myocardial infarction, mother | 1,168 (9.0%) | 746 (6.0%) | 1,914 (7.6%) | 0.113 |
| Register-based family history of myocardial infarction, mother | 1,578 (12.2%) | 1,478 (12.0%) | 3,056 (12.1%) | 0.007 |
| Self-reported family history of myocardial infarction, father | 2,889 (22.3%) | 2,377 (19.2%) | 5,266 (20.8%) | 0.075 |
| Register-based family history of myocardial infarction, father | 3,045 (23.5%) | 2,905 (23.5%) | 5,950 (23.5%) | 0.0004 |
| Self-reported family history of myocardial infarction, sibling | 387 (3.0%) | 222 (1.8%) | 609 (2.4%) | 0.078 |
| Register-based family history of myocardial infarction, sibling | 657 (5.1%) | 590 (4.8%) | 1,247 (4.9%) | 0.015 |
| Self-reported parental MI <60 years | 906 (7.0%) | 741 (6.0%) | 1,647 (6.5%) | 0.04 |
| Register-based parental MI <60 years | 461 (3.6%) | 444 (3.6%) | 905 (3.6%) | 0.002 |

*Categorical variables reported as n (%), continuous variables reported as medians (IQR). Abbreviations: CABG – coronary artery bypass grafting. IQR – interquartile range. MI – myocardial infarction. PCI – percutaneous coronary intervention.*

Supplementary Table S3. *Baseline characteristics for subjects excluded due to lacking coverage in the Swedish Multi-Generation Register.*

|  | Total |
| --- | --- |
|  | **N=4,690** |
| Female sex | 2,476 (52.8%) |
| Age | 57.50 (53.80-61.20) |
| Region of birth |  |
| Africa | 196 (4.2%) |
| Asia | 1,275 (27.2%) |
| EU15 outside of Nordics | 354 (7.5%) |
| Europe outside EU15 or Nordics | 1,341 (28.6%) |
| North America | 109 (2.3%) |
| Nordics other than Sweden | 628 (13.4%) |
| Oceania | 15 (0.3%) |
| Unknown | 2 (0.0%) |
| Post-Soviet states | 44 (0.9%) |
| Sweden | 476 (10.1%) |
| South America | 250 (5.3%) |
| Smoking |  |
| Former smoker | 1,610 (34.3%) |
| Never smoked | 1,949 (41.6%) |
| Current smoker | 843 (18.0%) |
| Missing | 288 (6.1%) |
| Education, highest completed level |  |
| Degree from university | 1,929 (41.1%) |
| History of myocardial infarction | 97 (2.1%) |
| History of PCI or CABG | 51 (1.1%) |
| Antihypertensive medication | 807 (17.2%) |
| Lipid lowering medication | 363 (7.7%) |
| History of diabetes | 282 (6.0%) |
| Self-reported family history of myocardial infarction, parent or sibling | 1,246 (26.6%) |
| Self-reported family history of myocardial infarction, mother | 399 (8.5%) |
| Self-reported family history of myocardial infarction, father | 815 (17.4%) |
| Self-reported family history of myocardial infarction, sibling | 224 (4.8%) |
| Self-reported parental MI <60 years | 352 (7.5%) |

*Categorical variables reported as n (%), continuous variables reported as medians (IQR). Abbreviations: CABG – coronary artery bypass grafting. IQR – interquartile range. MI – myocardial infarction. PCI – percutaneous coronary intervention.*

Supplementary Table S4. *Measures of accuracy of self-reported family history of myocardial infarction in parents and siblings, reported by females and males, respectively.*

| Reported by | Family history definition | Self-reported/register confirmed | | | | κ (95% CI) | Sensitivity (95% CI) | Specificity (95% CI) | Positive predictive value | Negative predictive value |
| --- | --- | --- | --- | --- | --- | --- | --- | --- | --- | --- |
|  |  | −/− | +/− | −/+ | +/+ |  |  |  |  |  |
| Females | MI at any age in parent or sibling | 7,403 | 1,011 | 1,685 | 2,854 | 0.527  (0.511 – 0.542) | 62.9%  (61.5% – 64.3%) | 88.0%  (87.3% – 88.7%) | 73.8%  (72.4% – 75.2%) | 81.5%  (80.6% – 82.3%) |
| Males |  | 7,240 | 793 | 2,069 | 2,247 | 0.453  (0.436 – 0.469) | 52.1%  (50.6% – 53.6%) | 90.1%  (89.5% – 90.8%) | 73.9%  (72.3% – 75.5%) | 77.8%  (76.9% – 78.6%) |
| Females | MI at any age in mother | 10,994 | 381 | 791 | 787 | 0.524  (0.500 – 0.548) | 49.9%  (47.4% – 52.4%) | 96.7%  (96.3% – 97.0%) | 67.4%  (64.6% – 70.1%) | 93.3%  (92.8% – 93.7%) |
| Males |  | 10,643 | 228 | 960 | 518 | 0.419  (0.393 – 0.446) | 35.0%  (32.6% – 37.5%) | 97.9%  (97.6% – 98.2%) | 69.4%  (66.0% – 72.7%) | 91.7%  (91.2% – 92.2%) |
| Females | MI <65 years in mother* | 12,132 | 119 | 65 | 84 | 0.470  (0.405 – 0.535) | 56.4%  (48.0% – 64.5%) | 99.0%  (98.8% – 99.2%) | 41.4%  (34.5% – 48.5%) | 99.5%  (99.3% – 99.6%) |
| Males |  | 11,812 | 96 | 81 | 69 | 0.431  (0.362 – 0.500) | 46.0%  (37.8% – 54.3%) | 99.2%  (99.0% – 99.3%) | 41.8%  (34.2% – 49.7%) | 99.3%  (99.2% – 99.5%) |
| Females | MI at any age in father | 8,944 | 964 | 1,120 | 1,925 | 0.545  (0.527 – 0.562) | 63.2%  (61.5% – 64.9%) | 90.3%  (89.7% – 90.8%) | 66.6%  (64.9% – 68.4%) | 88.9%  (88.2% – 89.5%) |
| Males |  | 8,631 | 813 | 1,341 | 1,564 | 0.483  (0.464 – 0.501) | 53.8%  (52.0% – 55.7%) | 91.4%  (90.8% – 91.9%) | 65.8%  (63.9% – 67.7%) | 86.6%  (85.9% – 87.2%) |
| Females | MI <55 years in father* | 11,955 | 287 | 56 | 102 | 0.361  (0.310 – 0.413) | 64.6%  (56.6% – 72.0%) | 97.7%  (97.4% – 97.9%) | 26.2%  (21.9% – 30.9%) | 99.5%  (99.4% – 99.6%) |
| Males |  | 11,625 | 260 | 70 | 103 | 0.372  (0.319 – 0.425) | 59.5%  (51.8% – 66.9%) | 97.8%  (97.5% – 98.1%) | 28.4%  (23.8% – 33.3%) | 99.4%  (99.2% – 99.5%) |
| Females | MI before age 55♂/ 65♀ in any parent* | 11,692 | 403 | 116 | 189 | 0.402  (0.361 – 0.443) | 62.0%  (56.3% – 67.4%) | 96.7%  (96.3% – 97.0%) | 31.9%  (28.2% – 35.8%) | 99.0%  (98.8% – 99.2%) |
| Males |  | 11,384 | 354 | 146 | 174 | 0.390  (0.348 – 0.433) | 54.4%  (48.7% – 59.9%) | 97.0%  (96.7% – 97.3%) | 33.0%  (29.0% – 37.1%) | 98.7%  (98.5% – 98.9%) |
| Females | MI <60 years in any parent | 11,875 | 617 | 172 | 289 | 0.394  (0.361 – 0.428) | 62.7%  (58.1% – 67.1%) | 95.1%  (94.7% – 95.4%) | 31.9%  (28.9% – 35.0%) | 98.6%  (98.3% – 98.8%) |
| Males |  | 11,419 | 486 | 189 | 255 | 0.404  (0.367 – 0.440) | 57.4%  (52.7% – 62.1%) | 95.9%  (95.5% – 96.3%) | 34.4%  (31.0% – 38.0%) | 98.4%  (98.1% – 98.6%) |
| Females | MI at any age in any sibling | 11,234 | 113 | 388 | 269 | 0.498  (0.460 – 0.535) | 40.9%  (37.2% – 44.8%) | 99.0%  (98.8% – 99.2%) | 70.4%  (65.6% – 75.0%) | 96.7%  (96.3% – 97.0%) |
| Males |  | 10,818 | 79 | 447 | 143 | 0.334  (0.291 – 0.376) | 24.2%  (20.8% – 27.9%) | 99.3%  (99.1% – 99.4%) | 64.4%  (57.7% – 70.7%) | 96.0%  (95.7% – 96.4%) |

*§Minus (–), absence of MI; plus (+) presence of MI
*Analysis performed in subset of subjects with no more than one self-reported family member afflicted by myocardial infarction.
Abbreviations: CI – confidence interval. MI – myocardial infarction.*

Supplementary Table S5. *Measures of accuracy of self-reported family history of myocardial infarction, by educational attainment.*

| Family history definition | University degree | Self-reported/register confirmed | | | | κ (95% CI) | Sensitivity (95% CI) | Specificity (95% CI) | Positive predictive value | Negative predictive value |
| --- | --- | --- | --- | --- | --- | --- | --- | --- | --- | --- |
|  |  | −/− | +/− | −/+ | +/+ |  |  |  |  |  |
| MI at any age in parent or sibling | Yes | 6,653 | 837 | 1,410 | 2,315 | 0.530  (0.513 – 0.547) | 62.1%  (60.6% – 63.7%) | 88.8%  (88.1% – 89.5%) | 73.4%  (71.9% – 75.0%) | 82.5%  (81.7% – 83.3%) |
|  | No | 7,990 | 967 | 2,344 | 2,786 | 0.462  (0.446 – 0.477) | 54.3%  (52.9% – 55.7%) | 89.2%  (88.5% – 89.8%) | 74.2%  (72.8% – 75.6%) | 77.3%  (76.5% – 78.1%) |
| MI at any age in mother | Yes | 9,664 | 260 | 682 | 609 | 0.519  (0.493 – 0.546) | 47.2%  (44.4% – 49.9%) | 97.4%  (97.0% – 97.7%) | 70.1%  (66.9% – 73.1%) | 93.4%  (92.9% – 93.9%) |
|  | No | 11,973 | 349 | 1,069 | 696 | 0.444  (0.420 –0.467) | 39.4%  (37.1% – 41.8%) | 97.2%  (96.9% – 97.5%) | 66.6%  (63.7% – 69.5%) | 91.8%  (91.3% – 92.3%) |
| MI <65 years in mother* | Yes | 10,650 | 97 | 38 | 65 | 0.485  (0.410 – 0.559) | 63.1%  (53.0% – 72.4%) | 99.1%  (98.9% – 99.3%) | 40.1%  (32.5% – 48.1%) | 99.6%  (99.5% – 99.7%) |
|  | No | 13,294 | 118 | 108 | 88 | 0.429  (0.368 – 0.491) | 44.9%  (37.8% – 52.1%) | 99.1%  (98.9% – 99.3%) | 42.7%  (35.9% – 49.8%) | 99.2%  (99.0% – 99.3%) |
| MI at any age in father | Yes | 7,849 | 824 | 946 | 1,596 | 0.542  (0.523 – 0.561) | 62.8%  (60.9% – 64.7%) | 90.5%  (89.9% – 91.1%) | 66.0%  (64.0% – 67.8%) | 89.2%  (88.6% – 89.9%) |
|  | No | 9,726 | 953 | 1,515 | 1,893 | 0.494  (0.477 – 0.511) | 55.5%  (53.9% – 57.2%) | 91.1%  (90.5% – 91.6%) | 66.5%  (64.7% – 68.2%) | 86.5%  (85.9% – 87.1%) |
| MI <55 years in father* | Yes | 10,474 | 255 | 39 | 82 | 0.347  (0.291 – 0.404) | 67.8%  (58.7% – 76.0%) | 97.6%  (97.3% –97.9%) | 24.3%  (19.8% – 29.3%) | 99.6%  (99.5% – 99.7%) |
|  | No | 13,106 | 292 | 87 | 123 | 0.381  (0.332 – 0.430) | 58.6%  (51.6% – 65.3%) | 97.8%  (97.6% – 98.1%) | 29.6%  (25.3% – 34.3%) | 99.3%  (99.2% – 99.5%) |
| MI before age 55♂/ 65♀ in any parent* | Yes | 10,279 | 350 | 72 | 149 | 0.397  (0.351 – 0.443) | 67.4%  (60.8% – 73.6%) | 96.7%  (96.4% – 97.0%) | 29.9%  (25.9% – 34.1%) | 99.3%  (99.1% – 99.5%) |
|  | No | 12,797 | 407 | 190 | 214 | 0.396  (0.357 – 0.435) | 53.0%  (48.0% – 57.9%) | 96.9%  (96.6% – 97.2%) | 34.5%  (30.7% – 38.3%) | 98.5%  (98.3% – 98.7%) |
| MI <60 years in any parent | Yes | 10,366 | 516 | 108 | 225 | 0.394  (0.357 – 0.432) | 67.6%  (62.2% – 72.6%) | 95.3%  (94.8% – 95.6%) | 30.4%  (27.1% – 33.8%) | 99.0%  (98.8% – 99.2%) |
|  | No | 12,928 | 587 | 253 | 319 | 0.402  (0.369 – 0.434) | 55.8%  (51.6% – 59.9%) | 95.7%  (95.3% – 96.0%) | 35.2%  (32.1% –38.4%) | 98.1%  (97.8% – 98.3%) |
| MI at any age in any sibling | Yes | 9,844 | 85 | 270 | 156 | 0.451  (0.404 – 0.499) | 36.6%  (32.0% – 41.4%) | 99.1%  (98.9% – 99.3%) | 64.7%  (58.3% – 70.8%) | 97.3%  (97.0% – 97.6%) |
|  | No | 12,208 | 107 | 565 | 256 | 0.410  (0.374 – 0.446) | 31.2%  (28.0% – 34.5%) | 99.1%  (99.0% – 99.3%) | 70.5%  (65.5% – 75.2%) | 95.6%  (95.2% – 95.9%) |

*§Minus (–), absence of MI; plus (+) presence of MI.
*Analysis performed in subset of subjects with no more than one self-reported family member afflicted by myocardial infarction.
Abbreviations: CI – confidence interval. MI – myocardial infarction.*

Supplementary Table S6. *Measures of accuracy of self-reported family history of myocardial infarction, by income.*

| Family history definition | Year and gender-adjusted income quintiles | Self-reported/register confirmed | | | | κ (95% CI) | Sensitivity (95% CI) | Specificity (95% CI) | Positive predictive value | Negative predictive value |  |
| --- | --- | --- | --- | --- | --- | --- | --- | --- | --- | --- | --- |
|  |  | −/− | +/− | −/+ | +/+ |  |  |  |  |  |  |
| MI at any age in parent or sibling | Q1 | 2,922 | 373 | 784 | 991 | 0.470  (0.444 – 0.496) | 55.8%  (53.5% – 58.2%) | 88.7%  (87.5% – 89.7%) | 72.7%  (70.2% – 75.0%) | 78.8%  (77.5% –80.2%) |  |
|  | Q2 | 2,909 | 354 | 772 | 1,025 | 0.487  (0.462 – 0.513) | 57.0%  (54.7% – 59.3%) | 89.2%  (88.0% – 90.2%) | 74.3%  (71.9% – 76.6%) | 79.0%  (77.7% – 80.3%) |  |
|  | Q3 | 2,896 | 355 | 771 | 1,038 | 0.490  (0.464 – 0.515) | 57.4%  (55.1% – 59.7%) | 89.1%  (88.0% – 90.1%) | 74.5%  (72.1% – 76.8%) | 79.0%  (77.6% – 80.3%) |  |
|  | Q4 | 2,931 | 361 | 719 | 1,049 | 0.507  (0.482 – 0.533) | 59.3%  (57.0% – 61.6%) | 89.0%  (87.9% – 90.1%) | 74.4%  (72.0% – 76.7%) | 80.3%  (79.0% – 81.6%) |  |
|  | Q5 | 2,985 | 361 | 708 | 998 | 0.502  (0.477 – 0.528) | 58.5%  (56.1% – 80.9%) | 89.2%  (88.1% – 90.2%) | 73.4%  (71.0% – 75.8%) | 80.8%  (79.5% – 82.1%) |  |
| MI at any age in mother | Q1 | 4,315 | 140 | 339 | 276 | 0.485  (0.446 – 0.524) | 44.9%  (40.9% – 48.9%) | 96.9%  (96.3% – 97.4%) | 66.3%  (61.6% – 70.9%) | 92.7%  (91.9% – 93.4%) |  |
|  | Q2 | 4,324 | 115 | 361 | 260 | 0.473  (0.434 – 0.513) | 41.9%  (38.0% – 45.9%) | 97.4%  (96.9% – 97.9%) | 69.3%  (64.4% – 74.0%) | 92.3%  (91.5% – 93.0%) |  |
|  | Q3 | 4,296 | 126 | 376 | 262 | 0.459  (0.420 – 0.499) | 41.1%  (37.2% – 45.0%) | 97.2%  (96.6% – 97.6%) | 67.5%  (62.6% – 72.2%) | 92.0%  (91.1% – 92.7%) |  |
|  | Q4 | 4,348 | 115 | 352 | 245 | 0.464  (0.424 – 0.505) | 41.0%  (37.1% – 45.1%) | 97.4%  (96.9% – 97.9%) | 68.1%  (63.0% – 72.8%) | 92.5%  (91.7% – 93.2%) |  |
|  | Q5 | 4,354 | 113 | 323 | 262 | 0.501  (0.460 – 0.541) | 44.8%  (40.7% – 48.9%) | 97.5%  (97.0% – 97.9%) | 69.9%  (64.9% –74.5%) | 93.1%  (92.3% – 93.8%) |  |
| MI <65 years in mother* | Q1 | 4,776 | 48 | 31 | 33 | 0.447  (0.345 – 0.549) | 51.6%  (38.7% – 64.2%) | 99.0%  (98.7% – 99.3%) | 40.7%  (29.9% – 52.2%) | 99.4%  (99.1% –99.6%) |  |
|  | Q2 | 4,769 | 46 | 32 | 39 | 0.492  (0.395 – 0.589) | 54.9%  (42.7% – 66.8%) | 99.0%  (98.7% –99.3%) | 45.9%  (35.0% – 57.0%) | 99.3%  (99.1% –99.5%) |  |
|  | Q3 | 4,769 | 49 | 36 | 28 | 0.380  (0.276 – 0.483) | 42.9%  (30.5% – 56.0%) | 99.0%  (98.7% – 99.2%) | 35.5%  (24.9% – 47.3%) | 99.3%  (99.0% – 99.5%) |  |
|  | Q4 | 4,802 | 34 | 28 | 26 | 0.450  (0.335 – 0.564) | 48.1%  (34.3% – 62.2%) | 99.3%  (99.0% – 99.6%) | 43.3%  (30.6% – 56.8%) | 99.4%  (99.2% – 99.6%) |  |
|  | Q5 | 4,828 | 38 | 19 | 28 | 0.490  (0.376 – 0.604) | 59.6%  (44.3% – 73.6%) | 99.2%  (98.9% – 99.4%) | 42.4%  (30.3% – 55.2%) | 99.6%  (99.4% – 99.8%) |  |
| MI at any age in father | | Q1 | 3,559 | 361 | 511 | 639 | 0.486  (0.457 – 0.515) | 55.6%  (52.6% – 58.5%) | 90.8%  (89.8% – 91.7%) | 63.9%  (60.8% – 66.9%) | 87.4%  (86.4% – 88.4%) |
|  |  | Q2 | 3,497 | 361 | 510 | 692 | 0.504  (0.475 – 0.532) | 557.6%  (54.7% – 60.4%) | 90.6%  (89.7% – 91.5%) | 65.7%  (62.8% – 68.6%) | 87.3%  (86.2% –88.3%) |
|  |  | Q3 | 3,494 | 344 | 489 | 733 | 0.532  (0.504 – 0.560) | 60.0%  (57.2% – 62.7%) | 91.0%  (90.1% – 91.9) | 68.1%  (62.5% – 70.8%) | 87.7%  (86.7% – 88.7%) |
|  |  | Q4 | 3,495 | 359 | 465 | 741 | 0.538  (0.510 – 0.565) | 61.4%  (58.6% – 64.2%) | 90.7%  (89.7% – 91.6%) | 67.4%  (64.5% – 70.1%) | 88.3%  (87.2% – 89.2%) |
|  |  | Q5 | 3,530 | 352 | 486 | 684 | 0.515  (0.486 – 0.543) | 58.5%  (55.6% – 61.3%) | 90.9%  (90.0% – 91.8%) | 66.0%  (63.0% – 68.9%) | 87.9%  (86.8% – 88.9%) |

*Table continues on the next page.*

*Table S6, continued.*

| Family history definition | | Year and gender-adjusted income quintiles | Self-reported/register confirmed | | | | κ (95% CI) | Sensitivity (95% CI) | Specificity (95% CI) | Positive predictive value | Negative predictive value |
| --- | --- | --- | --- | --- | --- | --- | --- | --- | --- | --- | --- |
|  |  |  | −/− | +/− | −/+ | +/+ |  |  |  |  |  |
| MI <55 years in father* | Q1 | 4,718 | 102 | 29 | 39 | 0.361  (0.277 – 0.445) | 57.4%  (44.8% – 69.3%) | 97.9%  (97.4% – 98.3%) | 27.7%  (20.5% – 35.8%) | 99.4%  (99.1% – 99.6%) |  |
|  | Q2 | 4,703 | 105 | 37 | 41 | 0.353  (0.271 – 0.434) | 52.6%  (40.9% – 64.0%) | 97.8%  (97.4% – 98.2%) | 28.1%  (21.0% – 36.1%) | 99.2%  (98.9% – 99.4%) |  |
|  | Q3 | 4,694 | 110 | 20 | 57 | 0.455  (0.377 – 0.534) | 74.03%  (62.8% – 83.4%) | 97.7%  (97.2% – 98.1%) | 31.4%  (27.0% – 41.9%) | 99.6%  (99.3% – 99.7%) |  |
|  | Q4 | 4,736 | 100 | 22 | 32 | 0.334  (0.246 – 0.422) | 59.3%  (45.0% – 72.4%) | 97.9%  (97.5% – 98.3%) | 24.2%  (17.2% –32.5%) | 99.5%  (99.3% – 99.7%) |  |
|  | Q5 | 4,729 | 130 | 18 | 36 | 0.316  (0.236 – 0.396) | 66.7%  (52.5% – 78.9%) | 97.3%  (96.8% – 97.8%) | 21.7%  (15.7% – 28.7%) | 99.6%  (96.4% – 99.8%) |  |
| MI before age 55♂/ 65♀ in any parent* | Q1 | 4,606 | 150 | 60 | 72 | 0.386  (0.320 – 0.452) | 54.5%  (45.7% – 63.2%) | 96.8%  (96.3% – 97.3%) | 32.4%  (26.3% – 39.0%) | 98.7%  (98.3% – 99.0%) |  |
|  | Q2 | 4,591 | 149 | 64 | 83 | 0.418  (0.354 –0.482) | 56.5%  (48.0% – 64.6%) | 96.9%  (96.3% – 97.4%) | 35.9%  (29.7% – 42.5%) | 98.6%  (98.2% – 98.9%) |  |
|  | Q3 | 4,585 | 158 | 53 | 85 | 0.425  (0.362 – 0.489) | 61.6%  (52.9% – 69.7%) | 96.7%  (96.1% – 97.2%) | 35.0%  (29.0% – 41.3%) | 98.9%  (98.5% – 99.1%) |  |
|  | Q4 | 4,648 | 134 | 50 | 58 | 0.369  (0.298 – 0.440) | 53.7%  (43.8% – 63.3%) | 97.2%  (96.7% – 97.6%) | 30.2%  (23.8% – 37.2%) | 98.9%  (98.6% – 99.2%) |  |
|  | Q5 | 4,646 | 167 | 35 | 65 | 0.374  (0.307 – 0.441) | 65.0%  (54.8% – 74.3%) | 96.5%  (96.0% – 97.0%) | 28.0%  (22.3% – 34.3%) | 99.3%  (99.0% – 99.5%) |  |
| MI <60 years in any parent | Q1 | 4,667 | 220 | 86 | 97 | 0.359  (0.303 – 0.414) | 53.0%  (45.5% – 60.4%) | 95.5%  (94.9% – 96.1%) | 30.6%  (25.6% – 36.0%) | 98.2%  (97.8% –98.6%) |  |
|  | Q2 | 4,639 | 212 | 83 | 126 | 0.432  (0.378 – 0.485) | 60.3%  (53.3% – 67.0%) | 95.6%  (95.0% – 96.2%) | 37.3%  (32.1% – 42.7%) | 98.2%  (97.8% – 98.6%) |  |
|  | Q3 | 4,649 | 218 | 81 | 112 | 0.399  (0.345 – 0.454) | 58.0%  (50.7% – 65.1%) | 95.5%  (94.9% – 96.1%) | 33.9%  (28.8% – 39.9%) | 98.3%  (97.9% – 98.6%) |  |
|  | Q4 | 4,658 | 225 | 62 | 115 | 0.418  (0.364 – 0.473) | 65.0%  (57.5% – 72.0%) | 95.4%  (94.8% – 96.0%) | 33.8%  (28.8% – 39.1%) | 98.7%  (98.3% – 99.0%) |  |
|  | Q5 | 4,681 | 228 | 49 | 94 | 0.380  (0.323 – 0.437) | 65.7%  (57.3% – 73.5%) | 95.4%  (94.7% – 95.9%) | 29.2%  (24.3% – 34.5%) | 99.0%  (98.6% – 99.2%) |  |
| MI at any age in any sibling | Q1 | 4,379 | 48 | 204 | 94 | 0.403  (0.344 – 0.462) | 31.5%  (26.3% – 37.2%) | 98.9%  (98.6% – 99.2%) | 66.2%  (57.8% – 73.9%) | 95.5%  (94.9% – 96.1%) |  |
|  | Q2 | 4,407 | 35 | 192 | 89 | 0.418  (0.357 – 0.479) | 31.7%  (26.3% – 37.5%) | 99.2%  (98.9% – 99.5%) | 71.8%  (63.0% – 79.5%) | 95.8%  (95.2% – 96.4%) |  |
|  | Q3 | 4,437 | 32 | 165 | 80 | 0.430  (0.365 – 0.495) | 32.7%  (26.8% – 38.9%) | 99.3%  (99.0% – 99.5%) | 71.4%  (62.1% –79.6%) | 96.4%  (95.8% – 96.9%) |  |
|  | Q4 | 4,410 | 47 | 144 | 87 | 0.457  (0.393 – 0.522) | 37,7%  (31.4% – 44.3%) | 98.9%  (98.6% – 99.2%) | 64.9%  (56.2% – 73.0%) | 96.8%  (96.3% – 97.3%) |  |
|  | Q5 | 4,419 | 30 | 130 | 62 | 0.421  (0.348 – 0.494) | 32.3%  (25.7% – 39.4%) | 99.3%  (99.0% – 99.5%) | 67.4%  (56.8% – 76.8%) | 97.1%  (96.6% – 97.6%) |  |

*§Minus (–), absence of MI; plus (+) presence of MI. *Analysis performed in subset of subjects with no more than one self-reported family member afflicted by myocardial infarction.
Abbreviations: CI – confidence interval. MI – myocardial infarction.*

Supplementary Table S7. *Age and birth year distributions of included relatives.*

| Type of relative | Median age of relatives* (IQR), years | Median birth year of relatives (IQR) |
| --- | --- | --- |
| Mother | 85 (80 – 91) | 1931 (1925 – 1937) |
| Father | 88 (83 – 94) | 1928 (1922 – 1934) |
| Sibling, full | 58 (53 – 64) | 1958 (1953 – 1964) |
| Sibling, half | 56 (47 – 66) | 1960 (1950 – 1969) |
|  |  |  |

**Corresponding age of relatives at the SCAPIS index visit, regardless of their vital status at that time.
Abbreviations: IQR – interquartile range.*

Supplementary Table S8. *Measures of agreement of self-reported family history of myocardial infarction in parents and siblings compared to register data, in subjects with the oldest parent born in 1930 or later.*

|  | Self-reported/register confirmed | | | | Cohen’s κ (95% CI) | Sensitivity (95% CI) | Specificity (95% CI) | Positive predictive value | Negative predictive value |
| --- | --- | --- | --- | --- | --- | --- | --- | --- | --- |
|  | −/− | +/− | −/+ | +/+ |  |  |  |  |  |
| MI at any age in parent or sibling | 7,017 | 1696 | 1,099 | 1,613 | 0.530  (0.511 – 0.549) | 59.5%  (57.6% - 61.3%) | 91.0%  (90.3% - 91.6%) | 69.9%  (67.9% - 71.7%) | 86.5%  (85.7% - 87.2%) |
| MI at any age in mother | 9,454 | 225 | 382 | 364 | 0.515  (0.481 – 0.548) | 48.8%  (45.2% - 52.4%) | 97.7%  (97.4% - 98.0%) | 61.8%  (57.5% - 65.7%) | 96.1%  (95.7% - 96.5%) |
| MI <65 years in mother* | 9,929 | 97 | 84 | 85 | 0.475  (0.410 – 0.540) | 50.3%  (42.5% - 58.1%) | 99.0% (98.8% - 99.2%) | 46.7%  (39.9% - 54.2%) | 99.2%  (99.0% - 99.3%) |
| MI at any age in father | 7,812 | 631 | 789 | 1,193 | 0.544  (0.523 – 0.565) | 60.2%  (58.0% - 62.4%) | 92.5%  (91.9% - 93.1%) | 65.4%  (63.2% - 67.6%) | 90.8%  (90.2% - 91.4%) |
| MI <55 years in father* | 9,731 | 222 | 114 | 128 | 0.416  (0.365 – 0.467) | 52.9%  (46.4% - 59.3%) | 97.8%  (97.5% - 98.1%) | 36.6%  (31.5% - 41.9%) | 98.8%  (98.6% - 99.0%) |
| MI before age 55♂/ 65♀ in any parent* | 9,470 | 317 | 193 | 215 | 0.432  (0.391 – 0.472) | 52.7%  (47.7% - 57.6%) | 96.8%  (96.4% - 97.1%) | 40.4%  (36.2% - 44.7%) | 98.0%  (97.7% - 98.3%) |
| MI <60 years in any parent | 9,424 | 395 | 291 | 315 | 0.444  (0.409 – 0.479) | 52.0%  (47.9% - 56.0%) | 96.0%  (95.6% - 96.4%) | 44.4%  (40.7% - 48.1%) | 97.0%  (96.6% - 97.3%) |
| MI at any age in any sibling | 9,579 | 38 | 155 | 88 | 0.468  (0.405 – 0.531) | 36.2%  (30.2% - 42.6%) | 99.6%  (99.5% - 99.7%) | 69.8%  (61.0% - 77.7%) | 98.4%  (98.1% - 98.6%) |

*§Minus (–), absence of MI; plus (+) presence of MI
*Analysis performed in subset of participants with no more than one self-reported family member afflicted by myocardial infarction.
Abbreviations: CI – confidence interval. MI – myocardial infarction*

References

1. Ekbom A. The Swedish Multi-generation Register. Methods Mol Biol. 2011;675:215-20. doi: 10.1007/978-1-59745-423-0_10.
2. Socialstyrelsen, The Swedish National Board of Health and Welfare. Statistical register's production and quality: National Patient Register. Stockholm: Socialstyrelsen; 2022.
3. Socialstyrelsen, The Swedish National Board of Health and Welfare. Statistical register's production and quality: National Cause of Death Register. Stockholm: Socialstyrelsen; 2022.
4. Brooke HL, Talbäck M, Hörnblad J, Johansson LA, Ludvigsson JF, Druid H, et al. The Swedish cause of death register. Eur J Epidemiol. 2017;32(9):765-73. doi: 10.1007/s10654-017-0316-1.
5. Johansson LA, Westerling R. Comparing Swedish hospital discharge records with death certificates: implications for mortality statistics. International journal of epidemiology. 2000;29(3):495-502. doi: 10.1093/ije/29.3.495.

1. Department of Clinical Science and Education, Södersjukhuset, Karolinska Institutet. Stockholm, Sweden [↑](#footnote-ref-1)
2. Institute of Environmental Medicine, Karolinska Institutet. Stockholm, Sweden [↑](#footnote-ref-2)
3. Department of Learning, Informatics, Management and Ethics, Karolinska Institutet. Stockholm, Sweden [↑](#footnote-ref-3)
4. Department of Medical Epidemiology and Biostatistics, Karolinska Institutet. Stockholm Sweden [↑](#footnote-ref-4)
5. Institute of Medicine, Sahlgrenska Academy, University of Gothenburg. Gothenburg, Sweden [↑](#footnote-ref-5)
6. Department of Medical Sciences, Clinical Epidemiology, Uppsala University. Uppsala, Sweden [↑](#footnote-ref-6)
7. Uppsala Clinical Research Center, Uppsala University. Uppsala, Sweden [↑](#footnote-ref-7)
8. Department of Clinical Sciences Malmö, Lund University. Malmö, Sweden [↑](#footnote-ref-8)
9. Department of Clinical Sciences, Danderyd University Hospital, Karolinska Institutet. Stockholm, Sweden [↑](#footnote-ref-9)
10. Department of Public Health and Clinical Medicine, Medicine, Umeå University. Umeå, Sweden [↑](#footnote-ref-10)
11. Department of Health, Medicine and Caring Sciences, Linköping University. Linköping Sweden [↑](#footnote-ref-11)
12. Centre for Medical Image Science and Visualization (CMIV), Linköping University, Linköping, Sweden [↑](#footnote-ref-12)
